# Supplementary material for: Calcium and Iron Content of Cereal-Based Gluten-Free Products
Source: Foods. 2022 Jul 6;11(14):2001. doi: 10.3390/foods11142001 (PMC9321653; doi:10.3390/foods11142001)
Supplement: Supplementary file 1 [file foods-11-02001-s001.zip › foods-1764828-supplementary.pdf]

Supplementary Table S1. Energy and nutrient data for individual gluten-free products per 100 g, inclusive of predominant flour types within the product.

		Energy (kcal)	Total fat (g)	SFA (g)	CHO (g)	Sugars (g)	Fibre (g)	Protein (g)	Iron (mg)	F	Calcium (mg)	F
	**BREAD: WHITE LOAVES**											
1	Maize, rice, millet	255	5.6	0.8	45	2.5	6.2	3.1	0.8		122	Y
2	Maize, rice, millet	255	5.6	0.8	45	2.5	6.2	3.1	-		119	Y
3	Maize, rice, rice	239	3.4	0.5	45	3.3	7.3	3.5	1.7		65	
4	Maize, rice, rice	239	3.4	0.5	45	3.3	7.3	3.5	2.4		107	
5	Rice, maize, buckwheat	226	3.0	0.3	41	5.8	5.5	4.8	2.1		65	
6	Rice, tapioca, maize	212	5.1	0.5	32	0.5	12.5	3.4	1.0		107	
7	Wholegrain rice	240	1.9	0.5	47	0.5	2.8	5.0	1.2		54	
8	White rice	221	2.8	0.2	44	0.1	1.7	4.1	1.0		110	Y
9	White rice, wholegrain rice	215	3.7	0.3	37	0.4	6.5	5.6	1.2		63	
10	White rice, millet	231	2.0	0.2	47	0.1	2.2	4.6	1.9		119	Y
11	Tapioca, potato, maize	236	6.1	0.5	36	2.2	5.5	4.0	1.7	Y	596	Y
12	Potato, maize, tapioca, rice, buckwheat	193	1.1	0.3	34	0.8	10.3	7.1	1.4		122	
13	Quinoa, wholegrain rice	215	2.8	0.4	38	2.2	3.2	6.8	1.5	Y	140	Y
	**BREAD: BROWN LOAVES**											
14	Maize, rice	233	4.3	0.7	41.0	4.5	7.4	3.8	2.8		118	Y
15	Maize, rice, tapioca	256	5.8	0.4	44.0	2.7	9.1	2.2	2.0	Y	188	Y
16	Maize, rice, quinoa	233	4.3	0.7	41	4.5	7.4	3.8	2.8		130	Y
17	Maize, rice, millet, quinoa	248	6.2	0.9	40	2.8	7.4	4.3	2.6		65	
18	Buckwheat, rice	210	1.8	0.2	42	0.4	6.2	5.0	1.9		95	
19	Wholegrain rice	244	4.8	0.6	41	0.6	4.5	5.7	1.6		71	
20	Potato, maize, rice sour dough	220	3.7	0.5	38	5.3	8.9	4.1	1.5		54	
	**BREAD: SEEDED LOAVES**											
21	Maize, rice, millet, quinoa	256	6.2	0.8	41	5.8	8.7	4.4	0.9		54	
22	maize, rice, millet, seeds	267	7.8	1.0	40	2.7	7.6	5.4	2.6		130	Y
23	Maize, rice, millet, seeds	267	7.8	1.0	40	2.7	7.6	5.4	2.1		125	Y
24	Maize, potato, tapioca, seeds	272	8.6	0.7	39	2.4	11	3.9	2.0	Y	200	Y
25	Potato, maize, tapioca, rice, seeds	241	7.2	1.0	28.5	0.6	14.2	8.5	1.3		35	

26	Maize, rice, seeds	262	9.2	1.1	36	0.9	8.8	4.5	2.3		71	
27	Maize, sweet potato	232	6.3	1.1	30.6	3.5	9.2	3.5	0.9		60	
	**BREAD ROLLS: WHITE**											
28	Maize, rice sour dough	231	3.3	0.6	42	3.9	6.8	4.6	**-**		60	
29	Maize, rice sough dough	261	3.2	0.5	51	4	7.5	3.3	0.9		48	
30	Maize, rice sough dough	219	2.6	0.5	41	4.9	8.1	3.9	2.1		48	
31	Maize, rice sough dough	267	3.0	0.5	54	6.3	6.4	2.9	1.3		65	
32	Maize, rice	248	3.0	0.2	49.5	2.7	4.9	2.8	1.1		107	
	**BREAD ROLLS: BROWN**											
33	Maize, rice sough dough	274	8.1	1	40	3.7	8.9	5.8	1.1		60	
34	Potato, maize, tapioca	238	5.6	0.5	34.9	2.3	7.1	8.4	1.3		18	
35	Maize, rice, seeds	278	8.0	1.1	41.3	5.7	8.2	6.2	1.1		83	
	**BAGUETTE: WHITE**											
36	Maize, rice sour dough	267	3.0	0.5	54	6.3	6.4	2.9	0.6		77	
37	Maize, rice	248	3.0	0.2	49.5	2.7	4.9	2.8	0.8		60	
38	Maize, rice	271	5.6	0.6	49	3.4	5.4	3.5	0.9		65	
39	Maize, rice	257	2.2	0.5	51.6	3.3	6.8	4.4	1.2		93	
	**BREAD: WRAPS n=11**											
40	Pea, rice, tapioca	306	8.0	0.9	37.1	3.5	9.0	17	0.9		88	
41	Rice, tapioca, maize	306	5.8	0.6	57	3.6	8.0	3.9	0.5		96	
42	Sorghum, rice, buckwheat	250	5.9	0.8	33.8	2.1	12.7	8.4	0.6		133	
43	Sweet potato, sorghum, rice	217	1.9	0.6	33	8.7	15.2	5.4	0.5		102	
44	Sorghum, maize, buckwheat	208	3.6	0.4	32	0.2	13	5.3	0.9		109	
45	Sorghum, maize, buckwheat	207	3.9	0.5	31	2.8	13	6.1	1.1		109	
46	Sorghum, maize, buckwheat	211	4.4	0.3	46	2.6	15	4.3	0.9		139	
47	Tapioca, maize	268	2.2	0.7	58	0.3	3.8	1.9	0.7		109	
48	Sorghum, maize, buckwheat	182	3.2	0.3	26.2	3.7	15.3	4.4	0.9		94	
49	Sorghum, maize, buckwheat	211	4.4	0.4	31	2.6	15.3	4.3	0.9		94	
50	Sorghum, chickpea, rice	211	4.4	0.4	31	2.6	15.3	4.3	0.9		127	
	**BREAD: PITTA**											
51	Rice, potato, maize	214	1.8	0.4	39.6	4.8	9.8	4.9	-		100	
52	Rice, tapioca, potato	229	5.1	0.4	37	4.4	12	2.6	0.8		112	
	**PIZZA BASE**											

53	Maize, potato, rice	294	4.8	0.6	58	5.4	4.6	2.3	0.6		100	
54	Maize, rice, tapioca	257	2.9	0.5	49.2	3.5	10	3.5	0.7		97	
	**NAAN**											
55	Tapioca, rice, maize	312	9.4	1	50	1.7	7.1	3.3	0.5		103	
56	Tapioca, rice	283	6.8	0.9	50	0.6	5.6	2.7	0.27		106	
57	Rice, tapioca	269	8	1	43	3	8	2.5	0.70		130	
	**PASTA**											
58	Maize, rice	355	1.8	0.6	76	0.4	2.3	7.5	1.3		60	
59	Maize, rice	157	0.5	0.1	35.3	0.5	0.5	3.1	0.3		65	
60	Maize, rice	357	1.3	0.4	78	0.2	1.4	7.4	1.1		71	
61	Rice, wholemeal rice, maize, quinoa,	170	1.2	0.4	36	0.5	0.9	3.3	0.3		42	
62	Rice, maize, sorghum, buckwheat…	349	1.5	0.5	72	4.4	6.5	8.5	1.2		119	
63	Green Pea	158	0.9	0.2	24.1	0.7	5.5	10.5	1.8		107	
64	Red Lentil	163	0.7	0.1	24.4	0.5	4.5	12.6	4		65	
	**CEREAL FLAKED**											
65	Rice, buckwheat, maize	380	2.7	0.5	75.8	12.7	7.8	9.2	0.8		89	
66	Maize,	400	3.3	0.5	80	13	10	10.0	0.6		130	
	**CEREAL BISCUIT**											
67	Sorghum	378	3.6	0.6	70	2.2	6.8	12.3	0.1		142	
	**MULTIPURPOSE FLOUR MIX**											
68	Rice, potato	338	2.3	1.4	72	5.8	6.2	4.1	4.8		119	Y
69	GF wheat starch, rice	350	0.5	0.3	80	11	2.4	5.2	1.2		402	Y
70	Rice, potato	359	1.1	0.9	82	3.5	3.1	3.6	0.3		54	
71	GF wheat starch, rice	331	1.3	0.2	69	6.2	12	4.7	0.3		296	Y
72	Rice, potato	343	0.5	0.2	76	10	6.3	5.5	1.6		414	Y
73	Maize	323	0.1	0	8.2	2.6	4.2	0.5	0.3		71	

Predominate flour type data, energy and macronutrient data for GF products from their labels. Calcium and Iron measured in the laboratory. SFA; saturated fatty acids, CHO; carbohydrate, F; fortified.
